# Dengue and Relative Bradycardia

**DOI:** 10.3201/eid1304.061212

**Published:** 2007-04

**Authors:** Aisha Lateef, Dale Andrew Fisher, Paul Ananth Tambyah

**Affiliations:** * National University Hospital; Singapore; † National University of Singapore, Singapore

**Keywords:** dengue fever, bradycardia, Singapore, letter

**To the Editor:** We have found that relative bradycardia is a notable clinical feature of dengue fever in Singapore. To our knowledge, this sign has not been previously associated with dengue. Awareness of this possible clinical finding could help in the early recognition of dengue and potentially reduce illness and death associated with dengue virus infection. Clinical features that can be used in the initial assessment of febrile patients are essential tools for clinicians, especially in limited resource settings.

Dengue fever is a potentially fatal illness; >2.5 billion persons are at risk and the disease is endemic in almost 100 countries (*1*). Singapore recorded >14,000 cases in 2005, its highest annual figure (*2*). No specific clinical features distinguish dengue from other febrile illnesses (*3*); thus, diagnosis relies heavily on results of laboratory investigations. Virus-specific immunoglobulin M (IgM) antibodies only become detectable after 5–7 days, and false-positive results can confound the diagnosis. PCR is a useful diagnostic tool; however, it is limited by the short duration of viremia and requirements for sophisticated laboratory support (*4*).

Relative bradycardia has been reported in many infectious diseases, including typhoid fever, Legionnaires’ disease, psittacosis, typhus, leptospirosis, malaria, and babesiosis (*5*,*6*). During the 2005 Singapore outbreak, we observed relative bradycardia in several patients with dengue fever. We therefore performed a case-control study comparing febrile dengue patients to patients with other infectious diseases. The study was approved by our hospital’s ethics committee.

The records of all patients admitted with a febrile illness to our general medical unit from June 1 to October 31, 2005, were reviewed. Patients with a clinical diagnosis of dengue fever and serologic confirmation (IgM or PCR positive) plus a temperature >38°C were included as case-patients. Age-matched controls were selected from the same general medical inpatient units and wereadmitted during the same period. All had fever, but they had a proven alternative diagnosis, including pneumonia (12 patients), upper respiratory tract infection (9 patients), urinary tract infection (6 patients), tuberculosis, liver abscess, viral fever other than dengue (3 patients each), meningitis, chicken pox, cellulitis, typhoid fever (2 patients each), and appendicitis, psoas abscess, typhus, infective endocarditis, pressure ulcers, and gastroenteritis (1 patient each). Exclusion criteria were the following: no laboratory confirmation, age <18 years or >60 years, preexisting substantial heart or lung disease or concurrent medication affecting heart rate, e.g., β-blockers, β-agonists, calcium channel blockers, or xanthine derivatives.

The peak temperature of all case-patients and controls was recorded within the first 24 hours of admission as well as heart rate and blood pressure at that point. Leukocyte count, hemoglobin concentration, hematocrit, and platelet counts were also noted. Data from 50 case-patients and 50 controls were tabulated and analyzed with Microsoft Excel (Microsoft Corp., Redmond, WA, USA).

The mean age (± standard deviation) for dengue patients was 32.8 (±10.8) years and for controls was 36.5 (±10.2) years (p = 0.08). There were 39 male patients in the dengue group and 31 in the control group. Their mean peak temperatures were comparable: 38.6°C (±0.5) (dengue) and 38.8°C (±0.7) (controls) (p = 0.09). Mean heart rates were significantly lower in the dengue group: 87.6 (±12.5) beats/min (dengue) and 104.6 (±14) beats/min (controls) (p<0.0001).

Electrocardiographs (ECGs) were available for 10 of the dengue group, and all showed normal sinus rhythm. Three patients with bradycardia had an ECG. Results for 2 patients were normal; 1 showed mitral valve prolapse with mild regurgitation. Ten patients in the control group underwent an ECG, and none had any notable abnormality. Four controls had ECGs, results for 2 were normal; 1 had mild mitral regurgitation, and 1 had mild tricuspid regurgitation. The heart rates at peak temperatures for patients with dengue fever were compared with rates for controls at all temperatures. Our findings demonstrate a consistently lower heart rate at all peak temperatures recorded (p<0.0001) (Figure).

**Figure:**
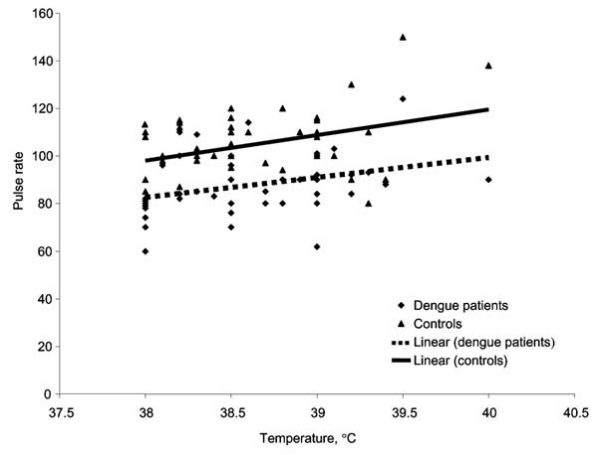
Temperature and heart rate relationship. Scatter plot for patients with dengue fever and nondengue febrile illness.

Dengue fever may adversely affect cardiac function. An echocardiographic study by Khongphatthanayothin et al. (*7*) showed depressed myocardial contractility and suboptimal heart rate response in some patients with dengue hemorrhagic fever. Acute reversible hypokinesia and reduction in left ventricular ejection fraction was also reported by Wali et al. (*8*). The underlying mechanisms were postulated to be immune in origin, although myocarditis may be a contributory factor. Fever production in response to exogenous pyrogens is believed to be mediated mostly by cytokine prostaglandin pathways, and neural input is important in the early phases of fever (*9*). Concentrations of cytokines, including tumor necrosis factor, interferon- γ, interleukin-8 (IL-8)**,** IL-10, and IL-12, are substantially increased during dengue infection. Their levels likely correlate with specific clinical manifestations and illness severity (*10*). The relationship of cytokines to relative bradycardia is unknown. Further studies could consider the relative importance of immune and neural mechanisms and also any direct cardiac pathology in the etiology of dengue-associated relative bradycardia.
